# The personality of newly graduated and employed nurses: Temperament and character profiles of Swedish nurses

**DOI:** 10.1016/j.ijnsa.2021.100058

**Published:** 2021-12-18

**Authors:** Marko Mihailovic, Danilo Garcia, Clara Amato, Erik Lindskär, Patricia Rosenberg, Elina Björk, Nigel Lester, Kevin M. Cloninger, C.Robert Cloninger

**Affiliations:** a Department of Psychiatry and Behavioral Sciences, Northwestern University, Chicago, IL, USA; b Promotion of Health and Innovation (PHI) Lab, International Network for Well-Being, USA; c Department of Behavioral Sciences and Learning, Linköping University, Linköping, Sweden; d Centre for Ethics, Law and Mental Health (CELAM), University of Gothenburg, Gothenburg, Sweden; e Promotion of Health and Innovation (PHI) Lab, International Network for Well-Being, Sweden; f Department of Psychology, University of Gothenburg, Gothenburg, Sweden; g Department of Psychology, Lund University, Lund, Sweden; h Promotion of Health and Innovation (PHI) Lab, International Network for Well-Being, Italy; i Anthropedia Foundation, St. Louis, Missouri, USA; j College for Public Health and Justice, Saint Louis University, St. Louis, Missouri, USA; k Center for Well-being, Washington University School of Medicine in St. Louis, St. Louis, Missouri, USA

**Keywords:** Character, Temperament, Nurses, Personality, Person-Centered Care, Resilience, Well-Being

## Abstract

**Background:**

One of the challenges of the 21st century is the high turnover rate in the nursing profession due to burnout and mental illness. From a biopsychosocial perspective, an individual's personality is an important vulnerability-resilience factor that comprises four temperament traits (i.e., a person's emotional reactions) and three character traits (i.e., self-regulation systems). Indeed, different personality profiles are associated to different coping strategies and health outcomes.

**Objective:**

We investigated and mapped the temperament and character of Swedish newly graduated and employed nurses’ in relation to the Swedish general population and an age-matched sub-sample.

**Design:**

In this cross-sectional study, nurses self-reported their personality (Temperament and Character Inventory) at the beginning of their employment.

**Setting:**

The data collection was conducted at a hospital in the South of Sweden.

**Participants:**

A total of 118 newly graduated and employed nurses (*M_ag_*_e_ = 25.95±5.58) and 1,564 individuals from the Swedish general population participated in the study.

**Methods:**

We calculated *T-scores* and *percentiles* for all seven personality dimensions using the Swedish norms (*N* = 1,564). The profiles were calculated by combining high/low *percentiles* scores in three temperament dimensions (Novelty Seeking: N/n, Harm Avoidance: H/h, and Reward Dependence: R/r) and in the three character dimensions (Self-Directedness: S/s, Cooperativeness: C/c and Self-Transcendence: T/t).

**Results:**

Regarding *T-scores*, the nurses reported moderately lower Novelty Seeking (> 0.5 *SD*), slightly higher Harm-Avoidance (about 0.5 *SD*), moderately higher Persistence (> 0.5 *SD*) and Reward Dependence (> 0.5 *SD*), and extremely lower Self-Directedness (> 1 *SD*). The prevalence of the most common temperament profiles among the nurses (Swedish general population in brackets) were: 39.80% [10.90%] Cautious (nHR), 21.20% [10.90] Reliable (nhR), and 15.30% [16.50%] Methodical (nHr). The prevalence of the most common character profiles among the nurses were: 31.40% [4.90%] Dependent (sCt), 25.40% [14.40%] Apathetic (sct), and 19.50% [8.80%] Moody (sCT).

**Conclusions:**

The analyses of the personality profiles showed that Low Novelty Seeking (79%), high Harm Avoidance (65%) high Reward Dependence (80%), low Self-Directedness (95%), and low Self-Transcendence (60%) were more prevalent among the newly graduated and employed nurses. This may partially explain newly graduated nurses’ difficulties at work and high turnover rate. After all, a well-developed character is of special importance when working with patients with serious and terminal illness or under large global crises, such as the current pandemic. Hence, both education at universities and development at work need to be person-centered to reduce stress levels and promote positive self-regulation strategies.

## Contribution of the paper

What is already known about the topic?

• There is a growing concern worldwide around well-being of nurses and the high turnover rate in the nursing profession.
• It is estimated that an additional of 9 million nurses are needed by 2030.
• There is uncertainty about what the causes behind this current global nurse shortage and what makes many nurses leave their career during their early years of employment.

What this paper adds

• This study is the first to investigate and map personality using the biopsychosocial model of personality in a sample of Swedish newly graduated and employed nurses.
• The biopsychosocial model of personality not only allows to investigate differences between individuals but also within individuals through the distinction of temperament and character dimensions.
• The results suggest that the newly graduated and employed nurses need stress-reducing interventions (e.g., 64% were high in Harm Avoidance) and tools for the promotion of healthy self-regulation strategies (95% were low in Self-Directedness).

Worldwide nurses are on the frontlines caring for the sick and injured and are expected to deliver high-quality patient care. During the COVID-19 pandemic, psychological distress and poor mental health including symptoms of anxiety and depression have been reported among healthcare workers. Nurses in particular, had a significantly higher incidence of severe depression compared with physicians (Lai et al., 2020). As the matter of fact, even before the pandemic the nursing profession was facing major challenges. Nurses and midwives represent 50% of the global health workforce and it is estimated that an additional of 9 million nurses will be needed by 2030 (WHO, 2020). At the same time, there is a high turnover rate in the nursing profession that has negative financial effects on hospitals and health care systems around the globe. For instance, the average cost of turnover for a bedside nurse in the United States ranged from $38,000 USD to $61,100 USD. On average, this nursing turnover cost a hospital between $4.4 million USD and $7 million USD extra in 2017 (NursingSolutions, 2018). This is a global concern because it poses a serious threat to patient safety, which becomes particularly important in face of the current and future challenges of the 21st century. Indeed, in addition to the expected turnover, the current shortage of workforce in the nursing field means that approximately 40 million new jobs in the health care industry will be required globally by 2030 (WHO, 2016). Hence, we need to focus on the processes of recruitment, but specially on the retention of workforce in the healthcare field (WHO, 2016).

According to available data, the number of newly graduated nurses has been increasing in recent decades, but the turnover rate has been high in most countries (Adhikari, 2015; Nursing, 2019). Thus, the high workforce shortage is a serious concern for patients, hospitals, and policymakers in the foreseeable future. Looking into global turnover rates, New Zealand had the highest (44.3%) followed by the United States (26.8%), Canada (19.9%), and Australia (15.1%) (Duffield et al., 2014)**.** A study conducted in Taiwan showed that 56.1% of 1283 surveyed nurses had intentions to leave (Lee et al., 2017) while 22.5% of 1137 surveyed nurses in China had intentions to leave within the following year (Jiang et al., 2017)**.** In Sweden, every fifth newly hired nurse reports strong intentions of leaving the profession (Rudman et al., 2014). Common reasons for nurses’ intention to leave their profession, besides aging, are low wages, high workloads, job stress, shift work, workplace culture, limited support systems, and difficult relationships with patients and physicians (Galletta et al., 2013; Takase, 2010). In addition, a shortage in the nursing field increases and adds up to the existing job stress, which in turn leads to burnout symptoms and job dissatisfaction (Galletta et al., 2013; Takase et al., 2015) An increased body of literature shows that turnover intention is spread worldwide among nurses and that it has a positive association with burnout and the lack of well-being (Havaei et al., 2016; Lagerlund et al., 2015; Takase et al., 2015)**.** In Sweden, 20% of newly graduated nurses report burnout symptoms in the first three years of their professional careers (Rudman and Gustavsson, 2011)**.** Among those with symptoms of burnout, the prevalence of intention to leave the profession was 27% after one year, 45% after three years, and 43% after five years of employment (Rudman et al., 2014). Thus, we need a better understanding of the factors that contribute to newly graduated nurses’ health and that allow them to stay in the profession despite all the challenges they face.

The transition from being a nursing student to becoming a professional nurse causes high levels of stress (Ashford and Nurmohamed, 2012; Saks and Gruman, 2011). Indeed, during the first years of nursing practice, approximately 20% to 50% of new registered nurses report stress-related symptoms (Boamah and Laschinger, 2016; Laschinger et al., 2010; Rudman and Gustavsson, 2011). Some studies indicate that nursing university programs are not consistent with real practice, thus, when becoming a professional nurse; nurses experience a “reality shock” which consist of high levels of stress (Rochester and Kilstoff, 2004; Ross and Clifford, 2002). Other studies even suggest that the lack of an adequate training and education in the nursing programs may result in self-esteem issues, which negatively influence newly graduated nurses when they step into the workplace and face the real challenges and demands of the profession (Clark and Holmes, 2007; Mooney, 2007). It has been suggested that focusing on the recruiting process could be a potential solution to the nursing shortage problem (Price et al., 2013)**.** However, the selection, recruitment, and retention of nursing students in nursing programs has also been a challenge and dropout rates of nursing students are high in most countries. For instance, 25% of nursing students in the United Kingdom and 40% in Canada do not finish their studies (Salamonson et al., 2014). In this context, applicants to nursing programs perceive that the nursing profession requires emotional stability and a resilient character in addition to knowledge, theoretical competence, adequate training, and general education (Glerean et al., 2019). Indeed, one of the main issues for mapping and understanding personality among helping professionals is that personality is one of the major factors for health and job retainment (Cloninger, 2004; Roberts et al., 2007).

While there is research on whether individuals with certain personality traits are drawn to or better suited to certain health care professions (Bore et al., 2009; Eley et al., 2012, 2015; Eley et al., 2013; Eley et al., 2016; D. Galarneau et al., 2021; McManus and Powis, 2007; Sievert et al., 2016), there is a lack of research that implements personality models that explain why certain personality profiles within newly graduated and employed nursers are more resilient than others. For instance, one of the consistently recognized reasons why nurses choose the profession is caring for others, which supports the widespread stereotype that nursing is a ‘caring profession’ (Williams et al., 2009); however, that does not explain if, when, and why being caring could be considered a vulnerability or a resilience factor for helping professionals. In this context, recent molecular research findings show that the basic unit of personality are profiles of temperament and character, not single traits (Cloninger and Zwir, 2018)**.** In other words, the study of personality needs to involve the complex dynamics between individual traits in order to understand how adaptation to internal and external conditions might lead to good health and resilience at work (Cloninger, 2004). Nevertheless, there is a lack of research addressing the intraindividual variability across personality profiles (Roberts and Hogan, 2001; Ryan and Sackett, 2012)**.**

The present study is intended to contribute to fill this gap by using the biopsychosocial model of personality developed by C. Robert Cloninger (Cloninger et al., 1993)**.** This personality model is appropriate for both between- and within-individual differences due to its distinction between nonintentional and intentional domains of personality, namely, temperament and character (Cervone, 2005)**.** In other words, using Cloninger's biopsychosocial model, we cannot only describe the personality of an individual, but also understand why an individual feels, thinks and acts as she/he does (Cervone, 2005).

## 1 The biopsychosocial model of personality

The biopsychosocial model of personality decomposes personality in two domains: temperament and character (Cloninger et al., 1993)**.** Temperament is responsible for emotional reactions such as anger, fear, disgust, and ambition (Cloninger, 2004, 2009; Cloninger, 2008)**.** There are four temperament traits (i.e., Novelty Seeking, Harm Avoidance, Reward Dependence, and Persistence) representing these unconscious emotional reactions that are related to procedural memory and that are relatively stable across the life span (Josefsson et al., 2013). Character, on the other hand, is related to our declarative memory system (semantic and episodic). It relates to our formed cognitive perspectives, responsible for the existence of individual differences in beliefs, goals, and values and the mental expressions of virtues such as hope, love, and faith (Cloninger, 2004, 2009; Cloninger, 2008). Character is formed and shaped through interactions with significant others as well as the environment and therefore more responsive to change through the life span (Cloninger et al., 2019)**.** There are three character traits: Self-Directness, Cooperativeness, and Self-Transcendence. These seven personality traits vary within individuals and across different populations (see Table 1 for a description of each temperament and character trait including sub-traits). Importantly, recent molecular research shows that both temperament and character have a large genetic etiology (roughly 50%), but that character is specifically related to genes that are behind epigenetic processes, that is, their phenotype can be activated through changes in the environment or interventions aimed directly at the character traits (Zwir et al., 2020a, 2020b; Zwir et al., 2019, 2021).

**Table 1:** Descriptors of high and low scorers on the Temperament and Character Inventory (TCI) subscales.

Personality Domain	TCI Scales	TCI Subscales	High Scorers	Low Scorers
**TEMPERAMENT**	**Novelty Seeking**	**NS1** excitability	exploratory	reserved
		**NS2** impulsivity	impulsive	rigid
		**NS3** extravagance	extravagant	thrift
		**NS4** disorderly	rule-breaking	orderly
	**Harm Avoidance**	**HA1** pessimism	pessimistic	optimistic
		**HA2** fearfulness	fearful	risk-taking
		**HA3** shyness	shy	outgoing
		**HA4** fatigability	fatigable	vigorous
	**Reward Dependence**	**RD1** sentimentality	sentimental	objective
		**RD2** openness	warm	aloof
		**RD3** attachment	friendly	detached
		**RD4** dependent	approval-seeking	independent
	**Persistence**	**PS1** eagerness	enthusiastic	hesitant
		**PS2** hard-working	determined	spoiled
		**PS3** ambition	ambitious	underachieving
		**PS4** perfectionism	perfectionistic	pragmatic
**CHARACTER**	**Self-Directedness**	**SD1** responsibility	responsible	blaming
		**SD2** purposefulness	purposeful	aimless
		**SD3** resourcefulness	resourceful	helpless
		**SD4** self-acceptance	unpretentious	pretentious
		**SD5** self-actualizing	self-actualizing	unfulfilled
	**Cooperativeness**	**CO1** social tolerance	tolerant	prejudiced
		**CO2** empathy	empathetic	self-centered
		**CO3** helpfulness	considerate	hostile
		**CO4** compassion	forgiving	revengeful
		**CO5** conscience	principled	opportunistic
	**Self-Transcendence**	**ST1** self-forgetfulness	engaged	self-concerned
		**ST2** transpersonal identification	joyfully connected, altruistic	separate individualistic
		**ST3** spiritual acceptance	faithful	skeptical
		**ST4** contemplation	contemplative	conventional
		**ST5** idealism	idealistic	cynical

Note: Adapted with permission from Anthropedia Foundation.

Previous studies (Eley et al., 2016; Lee et al., 2017; Stoyanov and Cloninger, 2012) have repeatedly shown that the temperament trait of Harm Avoidance and the character trait of Self-Directedness are important for determining one's susceptibility to psychopathology, resilience, well-being, and longevity (Cloninger, 2004; DeNeve and Cooper, 1998; Lee et al., 2014). More specifically, a high level of Self-Directedness is a critical protective factor against psychopathology and a factor in the promotion of mental health (Eley et al., 2013; Eley et al., 2016; Kim et al., 2013). On the other hand, high levels of Harm Avoidance are positively associated to maladaptive emotions (Aldao et al., 2010; Ball et al., 2002; Izadpanah et al., 2016; Manfredi et al., 2011; Peirson and Heuchert, 2001; Schreiber et al., 2012). Nevertheless, these “key traits” need to be examined in relation to the other temperament and character traits.

**Fig. 1:**
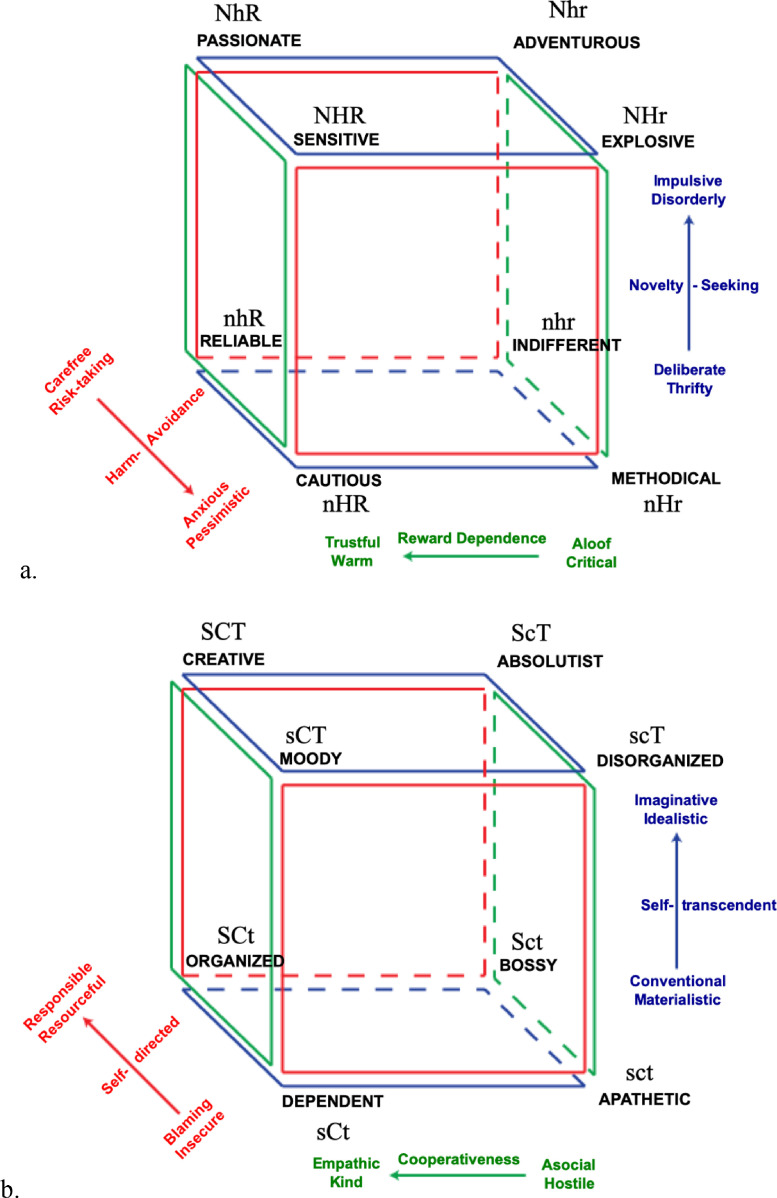
a, b. The temperament cube (a) representing the eight possible combinations of high and low scores in the temperament traits. The character cube (b) representing the eight possible combinations of high and low scores in the character traits. Note: Reprinted with permission from Anthropedia Foundation. High/low Novelty Seeking: N/n, high/low Harm Avoidance: H/h, high/low Reward Dependence: R/r; high/low Self-Directedness: S/s, high/low Cooperativeness: C/c, high/low Self-Transcendence: T/t.

Importantly, the theoretical and empirical differentiation between temperament and character makes it possible to discern differences within individuals (Cervone, 2005; Cloninger, 2004), thus, not only to describe people's personality but also explain why they have specific feelings, thoughts, and behaviors. Indeed, by considering personality as a complex adaptive system, it can be studied as combinations of temperament and character profiles (Cloninger, 2004; Kose et al., 2004)(Gusnard et al., 2003) (Cloninger et al., 2012; Gusnard et al., 2003). See Fig. 1a for the possible combinations of temperament interactions (i.e., the temperament cube) and Fig. 1b for those derived from character interactions (the character cube).

With regard to temperament profiles, individuals with a Reliable temperament profile (low Novelty Seeking, low Harm Avoidance, and high Reward Dependence) are considered as having a temperament with the highest levels of emotional maturity and stability, hence, with a high probability of developing a mature character (Cloninger, 2004). Indeed, individuals with this temperament are stable (low Novelty Seeking and low Harm Avoidance), warmly sociable (low Harm Avoidance and high Reward Dependence), and traditional (low Novelty Seeking and low Reward Dependence), thus, it is highly likely that they can be trusted to carry out what they are expected to do in a predictable and traditional manner (Cloninger, 2004). Individuals with a Passionate (high Novelty Seeking, low Harm Avoidance and high Reward Dependence), an Indifferent (low Novelty Seeking, low Harm Avoidance, and low Reward Dependence) or a Cautious (low Novelty Seeking, high Harm Avoidance, and high Reward Dependence) temperament profile are considered to have a low risk developing character immaturity and personality disorders. Nevertheless, there are some disadvantages with these profiles. For example, individuals with a Cautious temperament profile are inhibited (low Novelty Seeking and high Harm Avoidance), rejection-sensitive (high Harm Avoidance and high Reward Dependence), and traditional (low Novelty Seeking and high Reward Dependence). As a result, they have much social warmth (high Reward Dependence) and are likely to be careful and dutiful in carrying out responsibilities assigned to them (low Novelty Seeking). However, they have difficulty initiating anything new because of their inhibitions rooted in fears of rejection, criticism, loss, and change (high Harm Avoidance and high Reward Dependence). That being said, temperament alone does not determine whether a person is mature or immature and it is not directly associated with psychopathology (Cloninger, 2004).

With regard to character profiles, individuals with a Creative (high in all three character traits) or an Organized character profile (high in Self-Directedness and Cooperativeness but low in Self-Transcendence) consistently report the highest levels of well-being, healthy longevity, optimal cardiovascular health, including healthy lifestyle as well as reduced risk for chronic diseases. Having a Creative character profile is also linked with better heart rate variability or vagal tone in 24-hour recordings of heart rhythms (Zohar et al., 2013)**.** Individuals with a Creative profile tend to be constructive, keep things in perspective when faced with challenges (high Self-Directedness), they enjoy helping others, are compassioned (high Cooperativeness), and seek to grow in awareness of things that go beyond human existence (high Self-Transcendence). Conversely, individuals with an Apathetic (low in all three character traits) or a Disorganized character profile (low Self-Directedness, low Cooperativeness, and high Self-Transcendence) report the lowest levels of overall well-being (Cloninger, 2004)**.** Individuals with an Apathetic profile are not happy due to their experience of unhealthy emotions such as anxiety, alienation and have high rates of mental and physical disorders. Indeed, individuals with an Apathetic character profile feel victimized and helpless (low Self-Directedness and low Cooperativeness) and are injudicious (low Self-Directedness and low Self-Transcendence) and distrustful (low Cooperativeness and low Self-Transcendence). In other words, they experience the world from an outlook of separateness, which leads to fear, excessive desire, and false pride or self-reproach. Moreover, individuals with a Dependent character profile (low Self-Directedness, high Cooperativeness, and low Self-Transcendence) are submissive (low Self-Directedness and high Cooperativeness), injudicious (low Self-Directedness and low Self-Transcendence), and conventional (high Cooperativeness and low Self-Transcendence). Consequently, they depend on others to support and guide them, striving to conform to others’ expectations and traditions so as not to alienate the people that support them. This creates an insecure dependent relationship in which they are not self-reliant. A final example is people with the Moody character profile (low Self-Directedness, high Cooperativeness, and high Self-Transcendence), who are trustful (high Cooperativeness and high Self-Transcendence) but they are also submissive (low Self-Directedness and high Cooperativeness) and illogical (low Self-Directedness and high Self-Transcendence), and therefore experience a mix of positive and negative emotions that are unstable.

## 2 The present study

Since the biopsychosocial model of personality offers the possibility to test differences both between populations and within the individual through the assessment of personality profiles, we see this study as an important addition to the literature. We aimed to investigate and map the personality (i.e., temperament and character) of a sample of Swedish newly graduated and employed nurses in relation to the Swedish general population. To the best of our knowledge, this is the first time the biopsychosocial model of personality has been used in this endeavor.

### 2.1 Hypothesis

Since the nursing profession is a caring profession, we expected that:

1 the newly graduated and employed nurses would report high levels in the temperament trait of Reward Dependence compared to the Swedish general population.

Based on the high turnover rate and high levels of ill-being within newly graduated employed nurses reported in past literature, we expected:

2a that the nurses would report lower levels in the character traits, especially Self-Directedness, compared to the Swedish general population.
2b a low prevalence of the Creative and Organized character profiles among the nurses.

## 3 Method

### 3.1 Ethical statement

The study was approved by the Swedish Ethical Review Authority (Dnr. 2017/895) as part of a program between 2017 and 2020 targeting interventions aimed to prevent burnout and promote well-being in newly graduated and employed nurses in Blekinge, Sweden. The study was performed in accordance with the ethical standards of the 1964 Helsinki declaration and its later amendments. Participants were provided with the aims of the study, that participation was confidential and voluntary, that they had the opportunity to ask questions, and that they were free to withdraw at any time without giving a reason and without cost or any repercussions regarding their employment. Written consent was obtained from all participants.

### 3.2 Participants and design

All newly graduated employed nurses between the autumn of 2017 and the autumn of 2020 at the Blekinge Hospital were eligible for participation in this project (*N* = 242). All data was collected as part of the Hospital's introduction of newly graduated nurses who got employment in the Region of Blekinge, that is, about 2–3 weeks after the nurses’ first day of employment. In other words, to achieve our study's objective, we had access to the whole population of newly graduated nurses who got employment at the hospital during this specific period. A total of 162 nurses agreed to participate (67% response rate). However, only 118 participants had less than 5% missing data and were therefore the only ones included in the final analyses (i.e., 27% internal drop out and 51% total drop out). Nevertheless, the final data (*n* = 118) is roughly 50% of the whole population of the newly graduated nurses in Blekinge (*N* = 242), which is the number of responses required to achieve 5% margin of error at a confidence level of 95%.

### 3.3 Measure

#### 3.3.1 Temperament and character inventory (TCI)

For the measurement of the temperament and the character domains, the seven dimensions, and 16 profiles; Cloninger has developed the Temperament and Character Inventory (TCI) (Cloninger et al., 1993). This personality inventory is commonly used to measure personality viewed as a complex adaptive system in both clinical and normal populations. The TCI has been translated and psychometrically tested with both statistical and biological tools in 20 languages, including, French (Pelissolo et al., 2005), Swedish (Brandstrom et al., 2008)**,** Serbian (Dzamonja-Ignjatovic et al., 2010), Dutch (de la Rie et al., 1998)**,** Japanese (Kijima et al., 2000)**,** Turkish (Kose et al., 2004)**,** Bulgarian (Tilov et al., 2012)**,** and Spanish (Gutiérrez et al., 2001)**.** The normative data has been assessed and tested in more than 20 countries (e.g., the USA, France, Sweden, Serbia, Japan, China, Mexico, Italy, etc.) across four continents (North and South America, Europe, Asia, Oceania). The TCI has been shown to perform as well or better than other modern personality tests (Grucza and Goldberg, 2007; Picardi et al., 2005). Moreover, the TCI has been used to study the biological structure of personality using methods such as molecular neuroimaging (Borg et al., 2003)**,** structural neuroimaging (Yamasue et al., 2007)**,** and molecular genetics (Cloninger et al., 2019; Cloninger and Zwir, 2018; Cloninger and Cloninger, 2020; Zwir et al., 2019). Hence, we can now better understand the underlying biopsychosocial processes that influence the seven traits and the 16 personality profiles.

The TCI versions used here (the TCI-3) contain 240 items using a 5-point Likert scale (1 = *strongly disagree*, 5 = *strongly agree*). Some examples of the items measuring the four temperament traits are Novelty Seeking: “I often try new things just for fun or thrills, even if most people think it is a waste of time”, Harm Avoidance: “I often feel tense and worried in unfamiliar situations, even when others feel there is little to worry about”, Reward Dependence: “I like to discuss my experiences and feelings openly with friends instead of keeping them to myself”, and Persistence: “I usually push myself harder than most people do because I want to do as well as I possibly can”. Some examples of the items measuring the three character traits are Self-Directedness: “Each day I try to take another step toward my goals”, Cooperativeness: “I can usually accept other people as they are, even when they are very different from me”, and Self-Transcendence: “I sometimes feel so connected to nature that everything seems to be part of one living organism”.

### 3.4 Statistical treatment

We calculated *T-scores* (i.e., standardized scores were a score of 50 represents the *mean* of the general population and 10 points is equal to one *standard deviation*) and *percentiles* (i.e., the rank order of each individual in the general population) for the personality traits using the Swedish normative data (https://anthropedia.org; *N* = 1,564; see also Cloninger et al., 2021; Granjard et al., 2019, 2021). The percentiles were used to cluster participants in different temperament (high/low Novelty Seeking: N/n, high/low Harm Avoidance: H/h, high/low Reward Dependence: R/r) and character profiles (high/low Self-Directedness: S/s, high/low Cooperativeness: C/c, high/low Self-Transcendence: T/t). We mapped the *T-scores* and the prevalence of the temperament and character profiles among the nurses in relation to the whole Swedish general population and an age-matched sub-sample (*age range* = 21–50 years; *age mean* = 25.95±5.58) from the Swedish general population.

## 4 Results

A total of 118 participants (*age range* = 21–50; *age mean* = 25.95±5.58; 85 females, 10 males, and 23 missing gender data) had complete data on the personality variables (49% response rate). The *Cronbach's alphas* for the TCI in the present study were: 0.79 for Novelty Seeking, 0.92 for Harm Avoidance, 0.86 for Reward Dependence, 0.91 for Persistence, 0.89 for Self-Directedness, 0.87 for Cooperativeness, and 0.90 for Self-Transcendence.

**Fig. 2:**
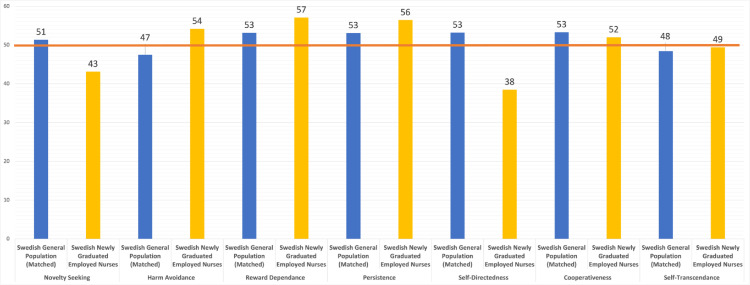
Showing T-Scores for each personality trait for the age-matched sample from Swedish general population (blue) and for the newly graduated employed nurses in our study (yellow). Note: The orange line marks the mean of the whole Swedish general population. A T-score of 50 represents the *mean* of the general population and 10 points is equal to one *standard deviation*.

**Fig. 3:**
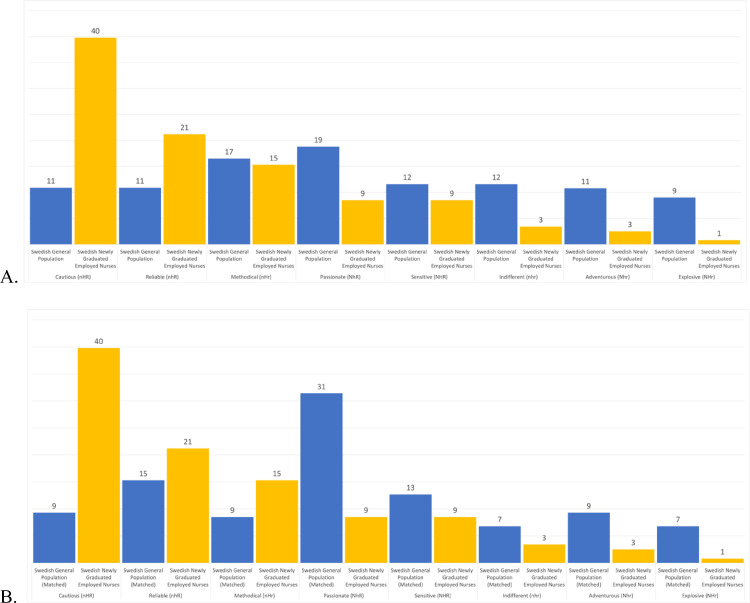
Showing the prevalence (%) of each temperament profile among the newly graduated employed nurses (yellow), (A) the whole Swedish general population (blue), and (B) the age-matched sub-sample from the Swedish general population (blue). Note: High/low novelty seeking: N/n, high/low harm avoidance: H/h, high/low reward dependence: R/r.

Regarding *T-scores* (see Fig. 2), compared to the whole Swedish general population, the newly graduated and employed nurses had similar levels of Cooperativeness and Self-transcendence, moderately lower levels of Novelty Seeking (> 0.5 *standard deviation* lower), slightly higher levels of Harm-Avoidance (about 0.5 *standard deviation* higher), moderately higher levels of Reward Dependence (> 0.5 *standard deviation* higher), moderately higher levels of Persistence (> 0.5 *standard deviation* higher), and extremely lower levels of Self-Directedness (> 1 *standard deviation* lower). These differences where almost the same when the nurses were compared to the age-matched subsample from the Swedish general population (see Fig. 2). The prevalence of temperament and character profiles among the newly graduated employed nurses, the Swedish general population, and the age-matched sample from the Swedish general population are shown in Figs. 3ab and 4ab.

**Fig. 4:**
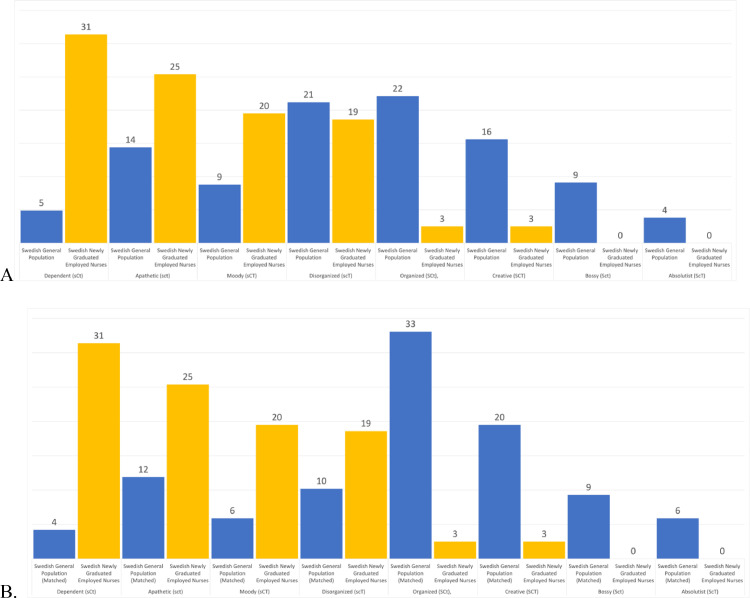
Showing the prevalence (%) of each character profile among the newly graduated employed nurses (yellow), (A) the whole Swedish general population (blue), and (B) the age-matched sub-sample from the Swedish general population (blue). Note: high/low self-directedness: S/s, high/low cooperativeness: C/c, high/low self-transcendence: T/t.

## 5 Discussion

The main aim of the present study was to explore personality (i.e., temperament and character) in a sample of Swedish newly graduated and employed nurses in relation to the Swedish general population. To the best of our knowledge, this is the first time the TCI has been used to map personality within a population of newly graduated nurses. At a general level (*T-scores* analyses), we found that nurses reported being reserved, orderly, and rigid (i.e., moderately low Novelty Seeking), prone to excessive worry, pessimism, doubt, and shyness (i.e., slightly high Harm Avoidance), that they are sympathetic, warm, sensitive, and approval-seeking (i.e., moderately high Reward Dependence), that they tend to not give up easily, quick to volunteer, and determined (i.e., moderately high Persistence), and report lacking purpose, self-acceptance and resourcefulness, as well as feeling ineffective and unreliable (i.e., extremely low Self-Directedness).

Moreover, to go beyond description of one's personality, we also mapped the personality profiles of the nurses. With regard to temperament profiles, the highest prevalence compared to the Swedish general population were the Cautious profile (i.e., low Novelty Seeking, high Harm Avoidance, and high Reward Dependence; nHR) with 29 and 31 percent points higher than the whole Swedish general population and the age-matched sub-sample; the Reliable profile (i.e., low Novelty Seeking, low Harm Avoidance, and high Reward Dependence; nhR) with 10 and 6 percent points higher than the whole Swedish general population and the age-matched sub-sample; and the Methodical profile (i.e., low Novelty Seeking, high Harm Avoidance, and low Reward Dependence; nHr) with similar prevalence than the whole Swedish general population and 6 percentage points higher than the age-matched sub-sample. Finally, regarding the character profiles, the highest prevalence compared to the Swedish general population were the Dependent profile (i.e., low Self-Directedness, high Cooperativeness, and low Self-Transcendence; sCt) with 26 and 27 percent points higher than the whole Swedish general population and the age-matched sub-sample; the Apathetic profile (i.e., low in all three character traits; sct) with 11 and 13 percent points higher than the whole Swedish general population and the age-matched sub-sample; and the Moody profile (i.e., low Self-Directedness, high Cooperativeness, and high Self-Transcendence; sCT) with 11 and 14 percent points higher than the whole Swedish general population and the age-matched sub-sample.

We expected nurses to have higher levels of Reward Dependence, since past research shows that caring for others is one of the main reasons individuals choose the profession (Williams et al., 2009). This temperament trait, we suggest, gives nurses the basis to warm communication with patients and colleagues, which is important for optimal patient care. In contrast to our findings, however, medical doctors, residents, and medical students report low levels of Harm Avoidance(Eley et al., 2016). This is important because there is a strong negative correlation between Harm Avoidance and both resilience and good health (Cloninger and Zohar, 2011; Eley et al., 2013; D. Galarneau et al., 2021; Josefsson et al., 2011). Moreover, since we were targeting a young population and because of the current high rates of turnover and burnout in the profession; we expected that nurses would report low levels of Self-Directedness—high Self-Directedness helps individuals to understand how personal actions influence outcomes expressed as responsibility, resourcefulness, self-acceptance, and the ability to admit and learn from mistakes. However, the extremely low levels of Self-Directedness among the nurses are alarming. Indeed, these levels were still extremely low even when compared to the age-matched sub-sample from the Swedish general population. In combination with high levels of Harm Avoidance and Reward Dependence, low levels of Novelty Seeking, and a stressful work climate, low levels of Self-Directedness might influence a warm, caring, reserved, and orderly nurse to feel victimized by the circumstances. In the long run, leading to burnout symptoms, leaving the profession, or what's even worse, long-term illness.

Converserly, previous findings show that along with high levels of Self-Directedness, medical students and physicians consistently report (Eley et al., 2009; Eley et al., 2016) high levels of Cooperativeness (i.e., being tolerant, empathetic, and compassionate). Within the health care profession, high levels of Cooperativeness would indicate that the individual has the ability to work well in interdisciplinary teams by establishing good quality relationships with their colleagues as well as patients based on high empathy, tolerance, and compassion—that is, social interaction not solely based on the motivation to seek approval from others or high Reward Dependence. Moreover, the development of Self-Transcendence (i.e., increasing in awareness that one is an inseparable part of a universal unity of being) has a radical transformative effect on high levels of both Self-Directedness and Cooperativeness (Cloninger, 2004, 2013): purposeful striving (i.e., high Self-Directedness) is transformed into hope and letting go of fighting and worry and tolerant empathy (i.e., high Cooperativeness) is transformed into love and working in the service of others. In other words, Self-Directedness, Cooperativeness, and Self-Transcendence should be important predictors of success in a demanding profession such as nursing. Hence, it is concerning that the nurses in our small sample reported extremely low levels of Self-Directedness and only normal levels of Cooperativeness and Self-Transcendence, since the character traits reflect personal maturity and are protective factors against burnout and necessary for the current and future challenges of the 21st century (Cloninger, 2013). If low character development is a vulnerability factor that is generally common among nurses, then it is plausible to suggest that being the ones in the frontline in combination with low Novelty Seeking, high Harm Avoidance, and high Reward Dependence might explain why nurses had a significantly higher incidence of severe depression compared with physicians during the COVID-19 pandemic (Lai et al., 2020).

That being said, nurses in the present study reported high levels of Persistence, which is consistent with findings from previous studies investigating the personality of medical professionals. High Persistence, the capacity to endure hard work and disappointment, is an important indicator of overall well-being but can also be expressed as perfectionism. Perfectionism is, for instance, a common characteristic of highly achieving individuals including medical professionals (Feinmann, 2011; Peters and King, 2012). It probably contributes to high quality control especially in conjunction with being highly orderly (i.e., low Novelty Seeking), thus, being important for all aspects of medical training and practice. However, there are advantages and disadvantages of high Persistence that depend on its interactions with other traits and between temperament and character profiles (Flett and Hewitt, 2002; Frost et al., 1990). Particularly, high Persistence in combination with low Harm Avoidance and high Self-Directedness, contributes to overall resilience. On the contrary, when high Persistence is combined with low Self-Directedness, as it is for our sample of nurses, it could contribute to susceptibility to stress and other unhealthy emotions such as anxiety and depression and even burnout, because the individual is perseverant, but lacks resourcefulness and self-acceptance, does not listen to bodily signals of stress and does not know when to let go (Cloninger et al., 2012; Ferguson et al., 2014).

Regarding the temperament profiles, the highest prevalence among the nurses, compared to the whole sample and the age-matched sub-sample from the Swedish general population, were the Cautious (nHR) and Reliable (nhR) profiles. Moreover, while the Methodical profile (nHr) was equally common among the whole Swedish population, it was more common among the nurses compared to the age-matched subs-ample from the Swedish general population. The Passionate (NhR), Sensitive (NHR), Indifferent (nhr), Adventurous (Nhr), and Explosive (NHr) profiles were less prevalent among the nurses compared to general population. Thus, most of the nurses (61%) reported being reserved, orderly, and rigid and at the same time being warm, attached, and approval seeking. Of this majority, some of the nurses were opposites in Harm Avoidance—40% high vs. 21% low: pessimistic vs. optimistic, fearful vs. risk-taking, shy vs. outgoing; fatigable vs. vigorous. Individuals with the most prevalent temperament profile among the nurses in the present study, the Cautious (nHR) profile, avoid doing anything that exposes them to risk of danger, rejection, and criticism, or avoid changing the way things are traditionally done. Hence, nurses with this profile have much social warmth (high Reward Dependence) and are likely to be careful and dutiful in carrying out responsibilities assigned to them (low Novelty Seeking), but they have difficulty initiating anything new because of their inhibitions rooted in fears of rejection, criticism, loss, and change (high Harm Avoidance and high Reward Dependence). This might lead to difficulties during organizational changes or even when daily routines change as a results of management decisions or global crises. The introduction and use of digital systems as new ways to provide care, for example, are an equal source of stress and distress for nurses who are starting their careers and for more experienced nurses (Kaihlanen et al., 2021; Yan et al., 2021). Hence, depending on their character profile, nurses having a Cautious (nHR) temperament profile might have difficulties to adapt to the digital future that has been speeded up by the current pandemic (Booth et al., 2021).

In essence, the temperament profile analyses showed that when the interactions within the individual were considered, about 80% of the nurses were warm, sentimental, friendly, and approval-seeking, that is, Cautious (nHR), Reliable (nhR), Passionate (NhR), or Sensitive (NHR); about 65% were pessimistic, fearful, and shy, that is, Cautious (nHR), Methodical (nHr), Sensitive (NHR), or Explosive (NHr); and about 79% were orderly, rigid, and reserved, that is, Cautious (nHR), Reliable (nhR), Methodical (nHr), or Indifferent (nhr). Depending on the temperament combinations, however, their reactions and motives to different circumstances might differ. For instance, a nurse with a Cautious (nHR) profile might have difficulties saying “no” due to high levels in both Reward Dependence and Harm Avoidance which make them fearful of social rejection, thus, feeling exploited by others. In contrast, nurses with a Reliable (nhR) profile are also driven by approval-seeking, but they are stable (low Novelty Seeking and low Harm Avoidance) and are highly likely to carry out what they are expected to do in a predictable and traditional manner and what is more important, they are more likely to have a mature developed character profile.

Regarding the character profiles, the highest prevalence among the nurses, compared to the whole sample and the age-matched sub-sample from the Swedish general population, were the Dependent (sCt), the Apathetic (sct), and the Moody (sCT) profiles; but to a certain degree also the Disorganized (scT) profile. The Organized (SCt), Creative (SCT), Bossy (Sct), and Absolutist (ScT) profiles were less prevalent among the nurses compared to Swedish general population. Thus, about 95% of the nurses reported being blaming, ineffective, and unreliable, that is, Dependent (sCt), Apathetic (sct), Moody (sCT), or Disorganized (scT); about 57% reported being tolerant, cooperative, and empathetic, that is, Dependent (sCt), Moody (sCT), Organized (SCt), or Creative (SCT); and about 60% reported being skeptical, cynical, and pragmatic, that is, Dependent (sCt), Apathetic (sct), or Organized (SCt). In this context, individuals with a Dependent (sCt) profile, the most common character profile among the nurses, require that others support and guide them, thus, they conform to others’ expectations and traditions so as not to alienate the people that support them. Individuals with an Apathetic (low in all three character traits; sct) profile, the second most common temperament profile among the nurses, report the lowest levels of overall well-being (Cloninger, 2004), because they experience the world from an outlook of separateness, which leads to fear, excessive desire, and false pride or self-reproach. Both profiles create insecure dependent relationships in which individuals are not self-reliant, thus, endangering the work climate and the care of patients. Indeed, one of the causes nurses leave the work force is difficult relationships with patients and physicians (Galletta et al., 2013; Takase, 2010).

Last but not the least, the combination of high Persistence, high Harm Avoidance, and low Self-Directedness, discerned in the present sample are typically over-achievers. Probably explaining past research suggesting that a majority of health care professionals are overly perfectionistic and compulsive; which makes them prone to burnout (Stoyanov and Cloninger, 2012) as well as predisposed to anxiety, depression, guilt, shame, pain hypersensitivity, and even suicide (Gabbard, 1985; Myers and Gabbard, 2008). However, these vulnerability factors are necessary but not sufficient to explain the high prevalence of burnout symptoms among nurses. According to the diathesis-stress model, for example, a vulnerable person might develop burnout symptoms as a response to a stressful psychological work climate and/or organizational culture (Stoyanov and Cloninger, 2012). Additionally, the high level of Reward Dependence in our sample of nurses is obviously a good trait for health professionals. However, depending on their temperament and character configuration, nurses who are high in Reward Dependence might have difficulty saying “no” to the demands of the profession, which could contribute to susceptibility to burnout in this population.

Furthermore, longitudinal data from Scandinavia shows that, except for Persistence which increases, the temperament dimensions stay steady on average; while both Self-Directedness and Cooperativeness increase from the age 25 to 35 (Josefsson et al., 2013). This indicates that the character development of the newly graduated and employed nurses in our sample lays ahead. However, their levels of Self-Directedness were extremely low even when compared to the age-matched sub-sample from the Swedish general population. Hence, time does not seem to be on our side. Therefore, interventions targeting stress reduction and character development need to be introduced during the nurses’ education and at the least during their first years of employment. For instance, in recent research conducted at the Blekinge Center of Competence, Sweden, in collaboration with the Anthropedia Foundation, we have shown that Well-Being Coaching (see https://anthropedia.org/well-being-coach-certification/) significantly increases subjective well-being and significantly decreases depression and anxiety among different populations, such as, unemployed young adults (Garcia et al., 2019) and refugees (Cloninger et al., 2021). What is more, Well-Being Coaching is easy to implement at an individual, institutional, or community level and in combination with mind-body interventions (i.e., modern forms of ancient SPA-practices for well-being promotion and stress reduction), it has shown synergetic effects and great promise in addressing the rising rates of stress, anxiety, and depression in the 21st century. Hence, increasing nursing students and newly graduated nurses' self-awareness and giving them tools that promote character development and calm their nervous system might have long-term effects on their health, resilience, and well-being (Campanella et al., 2014; Cloninger and Cloninger, 2011; Krasner et al., 2009).

## 6 Limitations and concluding remarks

The study was cross-sectional and with a relativity high dropout rate, thus, both longitudinal studies and larger samples are needed. Another plausible and important question is the choice of Cloninger's biopsychosocial model of personality as the basis of our study. After all, the Big Five (often measured using the Revised NEO Personality Inventory) is the personality model that is mostly used, especially in psychology. Nevertheless, Cloninger's biopsychosocial model and the TCI have been used since the 90′s and before that the temperament model has been used since the 80′s (see Cloninger, 2004) for the development history of the TCI). For example, a Google Scholar search using the keyword “Temperament and Character Inventory” gives approximately 87,000 hits and a search in the University of Gothenburg's library database for the same keyword gives approximately 14,000 hits. As discussed earlier, the TCI has been validated and translated into 20 different languages ​​including Swedish (Brändström et al., 1998), Serbian (Dzamonja-Ignjatovic et al., 2010), Dutch (de la Rie et al., 1998), Japanese (Kijima et al., 2000), Turkish (Kose et al., 2004) and Spanish (Gutiérrez et al., 2001). These and other studies show that all versions have sound psychometric properties that are comparable to the original English version. In Sweden specifically, researchers in different fields have used the model and different versions of the TCI among, for example, patients diagnosed with depression (Johansson et al., 2013), children and adolescents with and without different diagnoses and their parents (Anckarsäter et al., 2011), long-term unemployed (Granjard et al., 2019, 2021), refugees (Cloninger et al., 2021), and school children (Kapetanovic et al., 2020). It is important to point out that the “most accepted” model in personality psychology, the Big Five, was based solely on factor analytical studies (John and Roberts, 2021)—factor analytical methods without theory have previously been criticized (Block, 1995; Gould, 1981) and more recently psychologists accentuate the need of theoretically driven research (Proulx and Morey, 2021). However, we do not aim to convince the reader of the superiority of the TCI, we rather strive to show that there is sound logic behind the choice of model and instrument. After all, all paradigms have advantages and disadvantages, we have chosen what we consider the most complete personality model to date on basis of its theoretical and empirical distinction between temperament and character. From a theoretical point of view, the distinction of these two personality domains is based on how the human brain has evolved through evolution (Cloninger, 2004, 2009) and how different regions of the brain represent unconscious and conscious processes in different memory systems (Gazzaniga, 2011; Kahneman, 2011). Empirically, different molecular biological and neuroscientific studies have linked different neurotransmitters and brain systems to the different personality traits in Cloninger's biopsychosocial model (Yamasue et al., 2007). Cloninger's distinction between these two personality domains makes the model suitable for describing and understanding individual differences both within and between individuals (Cervone, 2005)—as opposed to the Big Five model which only measures stable temperament traits (McAdams, 2001) and can only describe the personality of an individual but not help us to understand why an individual feels, thinks and acts as she/he does (Cervone, 2005). In addition, independent researchers have compared the TCI with 11 of the most modern personality instruments. The TCI has demonstrated equal or higher predictive validity than these (Grucza and Goldberg, 2007). With all this said, there are also recent longitudinal genetic studies (i.e., genome-wide association study), using the TCI along with other types of data (e.g., parental reports, sociodemographic), that show that the temperament traits are associated with genes that are stable throughout life and that the character traits are associated with genes that are behind epigenetic processes (Zwir et al., 2020a, 2020b; Zwir et al., 2019). These findings were replicated in three different samples from three different countries (Germany, Finland, and Korea). In other words, meaning that temperament and character differ in whether these phenotypes can change or rather to what extent they can change due to their different genetic bases or etiology—the genes behind character, unlike the genes behind temperament, can be activated by external factors without the need to alter DNA (see also Josefsson et al., 2013) who in their longitudinal study show that temperament and character traits follow different kinds of developmental trajectories). A more recent genetic study, with an anthropological perspective, shows how character traits are linked to genes that are found in our ancestors, but that the genes associated to Self-Transcendence are only found in Homo Sapiens (Zwir et al., 2021). Self-Transcendence, for instance, is the personality trait that is the strongest predictor of overall personality change (Josefsson et al., 2013) and as discussed earlier, changes in Self-Transcendence have a major effect on both Self-Directedness and Cooperativeness.

Our study found that Swedish newly graduated and employed nurses differ in their personality when compared to the Swedish general population. The most marked differences were that the nurses in the present study had much higher levels of Reward Dependence and much lower levels of Self-Directedness. These differences were still present when the nurses were compared to the age-matched controls from the Swedish general population. Moreover, age-matched comparisons also revealed that the nurses were much lower in Novelty Seeking and had average levels in both Cooperativeness and Self-Transcendence. We have argued that the caring nature of the profession might explain why as much as 80% of the nurses were high in Reward Dependence when the temperament profiles where analyzed. In other words, explaining why individuals who are warm, caring, and approval-seeking choose becoming a nurse. Moreover, we suggested that low levels of character would be due to the young age of the nurses. However, even when compared with and an age-matched sub-sample of the Swedish general population, the nurses’ levels in Self-Directedness were extremely low. Hence, future studies need to address the question about self-selection of specific individuals into nursing education or if the nursing training has a specific effect on the personality development of nursing students. A pilot study in Sweden, for instance, demonstrated that third year nursing students reported lower levels of Self-Transcendence and higher levels of cynicism, a specific symptom of burnout, compared to first year nursing students (Björk, 2021).

Furthermore, one of the questions remaining is: How can these findings be used regarding the recruitment of nurses? This is a difficult question because, even though the use of personality tests is a common HR-practice for personnel selection, it is not without problems (Youngman, 2017). The ethical and practical implications on selecting nursing students or health care employees based on their general personality, or specific personality profile for that matter, are larger than we can discuss here. We argue, however, that relaying solely on the information gathered through a personality test is neither ethical or recommendable to identify the candidates who are best equipped to become nurses and to exclude the candidates least likely to succeed. Similarly, relying only on traditional recruitment methods, such as, in-person interviews, grades, resumes, IQ-tests, or references, is also precarious—all these methods risk the hiring of someone who is a false positive for becoming a quality nurse or rejecting someone who is a false negative. We think that the best and most ethical practice is to combine personality screening and traditional recruitment methods (Youngman, 2017) by cross-examining and mapping information congruency gathered by each one of these methods. However, the most important take home message is not about recruitment. Instead, our conclusions about newly graduated nurses having difficulties in their profession and life in general due to the specific personality issues detailed here, suggest that both basic nursing education and competence development at work need to be person-centered and aim to develop individuals’ character.

Last but not the least, the current global pandemic has shed light on nurses’ heroic efforts to mend the emotional, physical, and existential wounds of patients and their families worldwide. Nurses’ dedication, selflessness, and sacrifice add up to the existing and well-known challenges of the nursing profession. Previous studies have explored the prevalence of psychological outcomes among healthcare workers during infectious disease outbreaks (Chew et al., 2020, 2020; Kim, 2018; Lee et al., 2007; Su et al., 2006; Tan et al., 2020). For example, compared with those who did not participate in the care of SARS patients during the 2003 outbreak, healthcare workers reported sustained long-term effects even 1–2 years after the outbreak, such as, higher burnout, psychological distress, emotional exhaustion, anger, avoidance behavior, and post-traumatic stress disorder (Marjanovic et al., 2007; Maunder et al., 2006). Importantly, during the COVID-19 outbreak, high levels of family functioning, resilience (cf. low Harm Avoidance, high Persistence, and high Self-Directedness), and spirituality (cf. high Self-Transcendence) predicted two to six times lower probability of developing unhealthy emotions such as stress, anxiety, or depression (Kim et al., 2021)**.** Indeed, during the COVID-19 pandemic, psychological distress and poor mental health including symptoms of anxiety and depression have been reported among frontline healthcare workers. In other words, the COVID-19 pandemic has been a traumatic experience for many caring professions, but specially for nurses who had a significantly higher incidence of severe depression compared with physicians during this period (Lai et al., 2020). In this context, a recent study among workers in the New York area around the 9/11 terrorist attacks demonstrated that character development acts as buffer against the effects of this traumatic experience (Reynolds et al., 2021). More specifically, underdeveloped Self-Directedness and/or Cooperativeness were associated with incident post-disaster psychopathology, while the combination low Harm Avoidance, high Self-Directedness, and high Persistence was associated with post-disaster resistance to persistence and/or recurrence of preexisting psychiatric illness (Reynolds et al., 2021). Therefore, we argue that now more than ever, it is important to understand nurses’ personalities and their needs for character development during the current and future challenges of the 21st century. This might be seminal for nurses to provide good quality health care for those in need and stay resilient during these hard times.

## Declaration of Competing Interest

The authors declare that they have no competing interests. When this research was conducted, Danilo Garcia was the Head of Research of the Blekinge Center of Competence—Region Blekinge's research and development unit. The Center worked on innovations in public health and practice through interdisciplinary scientific research, community projects, and the dissemination of knowledge with the aim to increase the quality of life of the habitants of the county of Blekinge, Sweden. Danilo Garcia is also one of the founders and the Head of Research of the Promotion of Health and Innovation Lab at the International Network for Well-Being—a network of junior and senior researchers and students who are interested in the Science of Well-Being. Erik Lindskär is a research assistant at the Blekinge Center of Competence and Patricia Rosenberg was a project coordinator and a Well-Being Coach at the center. C. Robert Cloninger is the institute director and Kevin M. Cloninger is the CEO of Anthropedia Foundation. The Anthropedia Foundation is an educational non-profit organization that teaches individuals, professionals, and nonprofits ways to cultivate mental health and well-being in order to decrease rates of lifestyle- and stress-related illness.
